# Supplementation of Polymeric Reservoirs with Redox-Responsive Metallic Nanoparticles as a New Concept for the Smart Delivery of Insulin in Diabetes

**DOI:** 10.3390/ma16020786

**Published:** 2023-01-13

**Authors:** Vuk Uskoković

**Affiliations:** 1 TardigradeNano LLC, 7 Park Vista, Irvine, CA 92604, USA; vuk21@yahoo.com or vuk.uskokovic@tardigradenano.com or vuskokovic@sdsu.edu; 2 Department of Mechanical Engineering, San Diego State University, 5500 Campanile Drive, San Diego, CA 92182, USA

**Keywords:** composite, controlled release, diabetes mellitus, drug delivery, metallic nanoparticles, MnO_2_, PCL, thin film

## Abstract

Type 1 diabetes is caused by the inability of the pancreatic beta cells to produce sufficient amounts of insulin, an anabolic hormone promoting the absorption of the blood glucose by various cells in the body, primarily hepatocytes and skeletal muscle cells. This form of impaired metabolism has been traditionally treated with subcutaneous insulin injections. However, because one such method of administration does not directly correspond to the glucose concentrations in the blood and may fail to reduce hyperglycemia or cause hypoglycemia, the delivery of insulin in a glucose-dependent manner has been researched intensely in the present and past. This study tested the novel idea that the supplementation of polymeric reservoirs containing insulin with metallic nanoparticle precursors responsive to the redox effect of glucose could be used to create triggers for the release of insulin in direct response to the concentration of glucose in the tissue. For that purpose, manganese oxide nanoparticles were dispersed inside a poly(ε-caprolactone) matrix loaded with an insulin proxy and the resulting composite was exposed to different concentrations of glucose. The release of the insulin proxy occurred in direct proportion to the concentration of glucose in the medium. Mechanistically, as per the central hypothesis of the study, glucose reduced the manganese cations contained within the metal oxide phase, forming finer and more dissipative zero-valent metallic nanoparticles, thus disrupting the polymeric network, opening up pores in the matrix and facilitating the release of the captured drug. The choice of manganese for this study over other metals was justified by its use as a supplement for protection against diabetes. Numerical analysis of the release mechanism revealed an increasingly nonlinear and anomalous release accompanied by a higher diffusion rate at the expense of chain rigidity as the glucose concentration increased. Future studies should focus on rendering the glucose-controlled release (i) feasible within the physiological pH range and (ii) sensitive to physiologically relevant glucose concentrations. These technical improvements of the fundamental new concept proven here may bring it closer to a real-life application for the mitigation of symptoms of hyperglycemia in patients with diabetes.

## 1. Introduction

The attribute of smartness has been colloquially reserved for materials whose performance is responsive to the conditions of their environment. In the drug delivery field, this terminological umbrella has encompassed materials capable of releasing their pharmaceutical loads only upon experiencing a specific physical or chemical stimulus from the environment. Common examples include materials comprising disulfide bonds cleaving in response to the reductive intracellular thiol, glutathione [1,2], which cancer cells contain in greater abundance than normal ones [3]; polythiols swelling due to a sulfoxide → sulfone transition promoted under oxidative stress mediated by reactive oxygen radical scavengers and releasing entrapped drugs accordingly [4,5]; particles binding a drug through a hydrazone bond, which is stable under neutral conditions but breaks at an acidic pH [6,7]; thermosensitive hydrogels undergoing sol–gel transition at a critical temperature that is the function of the monomer ratio, molecular weight and its distribution, terminal functional groups and copolymer concentration [8]; endosomal escape of the drug load achieved by the selective dissolution of calcium phosphate nanoparticles as drug carriers inside acidic lysosomes [9,10,11]; alterations in the compactness and drug binding strength of chitosan particles as a function of pH due to the protonation/deprotonation of constitutive amine groups [12,13]; the shape memory effect, where the material revives its prior structure after severe deformation [14,15]; and others.

In this study, a novel concept for the smart delivery of insulin in diabetic patients is presented. Patients with diabetes tend to undergo erratic fluctuations in their blood glucose concentrations [16] because of either the impaired pancreatic insulin release process consequential to the loss of beta cells, as in Type I diabetes, or the inability of cells to respond to insulin normally, as in Type II diabetes. To stabilize the blood glucose levels, diabetics are prescribed insulin or, less commonly, an incretin hormone, such as glucagon-like peptide 1 [17], in one of many forms of delivery. Invariably, however, at least as far as commercial solutions are concerned, it is an injection because insulin is too large and hydrophilic of a molecule to be able to be absorbed through the oral route [18]. Still, numerous routes and modes of administration of insulin other than the subcutaneous have been investigated, ranging from oral to buccal to nasal to inhalable to intraperitoneal to transdermal [19].

As the ideal form of delivery of insulin would be tuned to the peaks and troughs of the glucose concentration in the bloodstream, lest the potentially dangerous conditions of hyperglycemia or hypoglycemia be encountered, the concept of the smart delivery of insulin has been studied intensely since the mid-2010s. The closest to the commercial domain that these smart delivery devices have come is as transdermal or epidermal patches that continuously monitor the blood glucose concentration in the interstitial fluid, saliva or sweat and administer insulin doses when the glucose levels exceed the typical hyperglycemic threshold in the blood of around 4 mg/mL. The majority of such systems on the market and in clinical studies are electromechanical devices employing closed-loop continuous glucose-monitoring sensors and external insulin infusion pumps. Systems relying on glucose-responsive chemistries, on the other hand, have yet to make a clinical and commercial breakthrough, despite numerous notable mentions. In a study by Yu et al. from 2015, for example, insulin and glucose peroxidase were encapsulated inside hyaluronic vesicles, creating hypoxic conditions due to oxidation of glucose via glucose peroxidase, which led to the reduction of nitroimidazole groups on the vesicles into hydrophilic aminoimidazoles, dissociating the vesicles and releasing insulin [20]. The following year, Zou et al. reported on mesoporous silica nanoparticles functionalized with alizarin complexone, which itself was bound to insulin using a benzene-1,4-diboronic acid-mediated esterification reaction, so the presence of glucose caused the dissociation of the boronate ester and the release of insulin [21]. Chang et al. next reported on nanoparticles co-assembled from concanavalin A and amylopectin, where the affinity of the former component for sugars was so high that the nanoparticles disassembled upon the addition of glucose and released their insulin load [22]. Chen et al. were the next to synthesize an acrylamide hydrogel combined with a phenylboronic acid derivative, which exhibited a glucose-dependent shift between uncharged and negatively charged boronate ions, leading to corresponding changes in the hydration state and thus in the insulin release rates [23]. In parallel with these studies, a decent number of them have accomplished a similar glucose-responsive release effect, but without employing an equally rational mechanistic design. 

**Figure 1:**
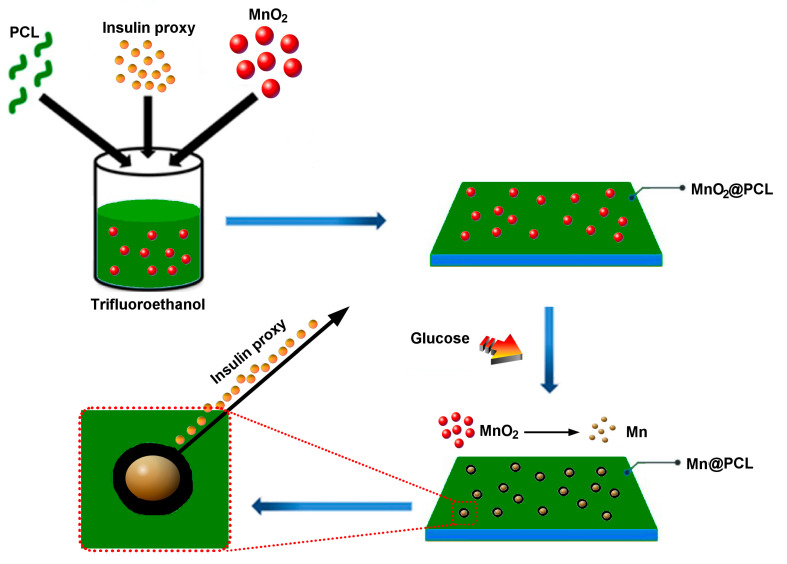
Schematic description of the concept behind the smart delivery of an insulin proxy from PCL thin films supplemented with MnO_2_ as a redox-responsive metallic nanoparticle precursor. PCL, the insulin proxy and MnO_2_ nanoparticles were mixed with 2,2,2-trifluoroethanol and cast inside a Petri dish. Once solidified, the film was exposed to glucose, which prompted the reduction of MnO_2_ nanoparticles and their partial conversion to metallic Mn, leaving behind pores acting as channels for the release of the insulin proxy.

The pivotal premise of this study was that the supplementation of polymeric reservoirs containing insulin with metallic nanoparticle precursors responsive to the redox effect of glucose could be used to create triggers for the release of insulin in direct response to the concentration of glucose in the tissue. This concept is illustrated in Figure 1. Specifically, according to this concept, a cast layer of polymer is being supplemented with metal oxide nanoparticles, which convert partially to zero-valent metallic nanoparticles upon exposure to reductive glucose, altering the polymeric network locally and facilitating the release of insulin molecules captured within the polymeric matrix. Glucose is a relatively potent green reduction agent owing to the presence of the aldehyde group, which undergoes oxidation to form carboxylic acids, namely gluconic and glucaric acids [24], during the process of which reactive reagents can be reduced by capturing electrons released by oxidized glucose. In fact, preparations of numerous metallic nanoparticles under ambient or near-ambient conditions, ranging from copper [25] to silver [26] to gold [27] to platinum [28], have successfully utilized glucose as a reduction agent, thus circumventing the standard usage of more reactive, but also less environmentally friendly, chemicals, such as borohydrides or hydrazine. 

To test this new concept, numerous materials were considered as candidates for the polymeric carrier of the model drug acting as the proxy for insulin. Polymers lying on the high end of the degradation and release rate spectrum were discarded as an option because they would make it exceedingly difficult to observe the presumably minor effects of the redox reaction of the metallic nanoparticle precursors. For this reason, the choice fell on poly(ε-caprolactone) (PCL), a polymer that is biodegradable, but with a relatively long residence time in the body, typically in the order of months or even years [29]. 

PCL is a biocompatible polymer that has been used for a number of applications as a biomaterial, including as a drug delivery carrier [30]. Its biodegradability is mainly spontaneous, occurring via the hydrolytic scission of the ester linkages, but can also be enzymatically reinforced, in which case the degradation can be considerably sped up, from taking 1–2 years when relying on mere hydrolysis by water molecules [31] to a matter of weeks in an environment favoring enzymatic digestion [32]. Unlike polymers such as polyvinyl alcohol, polyvinyl chloride or polysiloxane, which have all been known to provoke inflammatory reactions or fibrous tissue formation in the body [33,34], PCL has been deprived of these issues. The degradation products of PCL [35], like lactic and glycolic acids resulting from the biodegradation of poly(lactic-co-glycolic acid), can be incorporated into the body’s natural metabolic cycles in the course of the degradation of the polymer. Some of these degradation products include butyric acid, which is one of the most common short-chain fatty acids naturally present in the mammalian gut; caproic acid, which is naturally present in dairy, bread, beer and other food; valeric acid, which is a natural minor product of the gut microbiome; and succinic acid, which is a molecule with multiple biological roles, ranging from acting as a metabolic intermediate in the electron transport chain to acting as a signaling molecule. Because of this, PCL has been used as a biomaterial not only for external applications, such as wound dressings [36], but also for internal ones, including as a base for the fabrication of tissue-engineering constructs [37] or implantable drug delivery carriers [38]. 

As for the redox-responsive metallic nanoparticle precursors as candidates for the inorganic component of the composite material proposed, there could be many, with the most commonly performed synthesis of zero-valent metallic nanoparticles being that of gold from HAuCl_4_ as the precursor [39]. Gold also comprises a convenient substrate for the non-enzymatic sensing of glucose by surface-enhanced Raman scattering [40]. However, instead of gold, which is completely foreign to the human body, a better solution is thought to involve the use of one of many trace metallic elements that are deficient in patients with diabetes, be it Type 1 or Type 2. Such metals include chromium, manganese, cobalt, copper, and zinc [41]. Some of these metals, such as chromium, especially in its hexavalent form, or cobalt, can be very toxic, but others may present viable choices for testing. Here, in this proof-of-concept study, the choice fell on manganese, given that using this element as a supplement has been shown to have a protective effect against diabetes [42]. 

The use of water-soluble manganese salts as redox-reactive metallic nanoparticle precursors was considered ineffective because of their tendency to be dissolved from the polymer prior to having the chance to form zero-valent particles in the matrix of the polymer and disturb its structure. Such salts would be released from the polymer before reacting with glucose and the products of the reduction reaction might also precipitate over the fibers, thus even possibly blocking the release of the drug. To achieve the formation of metallic nanoparticles within the polymeric matrix, which would open up the polymeric structure and increase the water influx and the degradation rate, the use of insoluble precursors is needed. In this study, manganese oxide (MnO_2_) was selected as one such insoluble precursor, which would form wholly or partially metallic Mn nanoparticles upon interaction with glucose. Oxides are generally easier to reduce than carbonates or sulfides, explaining the choice of MnO_2_ as the precursor. Additionally, there are common methods for synthesizing MnO_2_ nanoparticles [43,44,45], one of which was adapted for the purposes of this study [46]. The reason for resorting to fine, nanosized particles of MnO_2_ is that the intended reduction reaction is expected to proceed more efficiently when the contact area between the precursor and the reductant is high, which would correspond to precursor particles of a small size and high surface-to-volume ratios. Prior studies have shown that environmentally friendly reduction agents, including ascorbate [47] and glucose [48], amongst others, are effective in reducing divalent metal oxides to pure metallic nanoparticles, with the only caveat being that the reduction reactions often proceed only to partial completion [49], even with the employment of elevated temperatures or hydrothermal conditions. In addition, more than a century ago, it was recognized that glucose is not spontaneously oxidized by air in aqueous solutions and that alkaline conditions, such as those achieved by the addition of disodium phosphate [50] or an even stronger base, e.g., sodium hydroxide [51], are necessary for this reaction to proceed [52]. Another observation made over a century ago is that electron transfer reactions between glucose and MnO_2_ are possible under alkaline conditions, leading to a series of structural transformations in the oxide phase [53], which were attempted to be harnessed for drug delivery purposes in this study. 

At around the same time, in analogy with the fact that iron and manganese species play a key role in oxidation reactions in vivo, it was shown that both of these ions, but especially iron, catalyzed the oxidation of glucose by air in aqueous solutions [54,55], further explaining the decision to choose Mn as a redox-reactive element in this study. The ability to oxidize glucose, in fact, was used to justify the proposition for the use of manganese oxide (Mn_x_O_y_) nanoparticles as facilitators of the starvation of cancer cells [56]. Manganese, moreover, is an element that cycles across the ecosphere in its various redox states, such as in deep-sea basins, where dissolved Mn^2+^ and intermediate Mn^3+^ species are transported upward by diffusion until they are oxidized into Mn^4+^ and form MnO_2_, which is solid and is transported downward due to gravity, toward anaerobic depths where it then undergoes reduction by sulfides and continues cycling [57]. This omnipresence of manganese redox processes and their essentiality for life provides strong support for choosing this element for incorporation into the smart drug delivery carrier investigated here. To reiterate, the central hypothesis underlying the new concept for the smart delivery of insulin proposed here is that the exposure of MnO_2_ nanoparticles interspersed across a polymeric network to glucose would trigger the reduction of the metal ions and their partial conversion to metallic Mn, leading to perturbations of the polymeric structure and the release of insulin from it, the amount of which would be directly proportional to that of glucose. The concept of using the reduction of metal oxides embedded in a polymeric drug carrier via glucose or any other reductants to facilitate the release of the drug is novel and has not been proposed or empirically tested yet, to the best of the author’s knowledge. 

## 2. Materials and Methods

Manganese oxide (MnO_2_) nanoparticles were synthesized by adding 100 mL of an aqueous solution of 2 M NaOH dropwise to the same volume of vigorously stirred 1M MnSO_4_ solution. The solution was aged at 60 °C for 2 h to ensure the completion of the reaction. The precipitate was separated from the parent solution by centrifugation and washed trice with deionized water. The washed precipitate was dried in an oven at 100 °C overnight. The thus-prepared MnO_2_ nanopowder was mixed at a concentration of 10 wt.% with poly(ε-caprolactone) (PCL, M_w_ = 80–100 kDa) and methyl orange (Sigma-Aldrich, St. Louis, MO, USA) as the insulin proxy in 2,2,2-trifluoroethanol. PCL and mildly hydrophobic methyl orange (logP_oct/water_~0.15 at the neutral pH) were dissolved in trifluoroethanol, while MnO_2_ particles were dispersed in it under mild agitation for 3 h at 65 °C. The resulting suspension was poured into Petri dishes and the solvent was allowed to evaporate under ambient conditions. The resulting inorganic/organic composite films were peeled off the glass surface with forceps and used for the drug release analyses.

Drug release was measured by immersing precisely weighed pieces of the composite MnO_2_@PCL film (1 g) loaded with methyl orange as the insulin proxy in 10 mL Eppendorf tubes containing phosphate-buffered saline set to pH 10 with the addition of NaOH and supplemented with 0.05 vol.% Tween 80 so as to create sink conditions for methyl orange. To prevent the segregation of the latter compound in a hydrophilic medium and facilitate its homogeneous distribution across the volume of the release solution, the solution was amended with a surfactant that would break down any drug molecule aggregates and solubilize the constitutive molecules, namely Tween 80. Different concentrations of D-glucose anhydrous (C_6_H_12_O_6_, Santa Cruz Biotech. Co., Dallas, TX, USA) were also added to dedicated sample groups so as to compare the effect of glucose on the release of methyl orange, including 0, 100, 200 and 300 mg/mL, which corresponded to 0, 0.55, 1.11 and 1.67 mM, considering the M_w_ of 180.1 g/mol for glucose. The release vials were mildly agitated in an orbital shaker throughout the release process and the temperature was maintained at 37 °C. One milliliter of the medium was collected after specific periods of time and analyzed for absorption at a wavelength of 464 nm on a UV/Vis spectrophotometer (Nanodrop 2000, Thermo Scientific, Waltham, MA, USA), while the same volume of the aliquoted buffer was replaced with the fresh buffer supplemented with a fresh dose of glucose. All the data points in the drug release diagrams represent averages of experimental triplicates (n = 3), while the error bars represent the standard deviation (SD = (Σ(x − $\bar{x}$)^2^/n)^1/2^). Data analysis was performed in Microsoft Excel and OriginLab OriginPro 2018. GraphPad QuickCalcs unpaired *t*-test calculator was used to measure the statistical significance of the difference between the values of individual sample groups. A confidence level of *p* < 0.05 was considered statistically significant. 

The drug release profiles in the entire release range were fitted to the Korsmeyer–Peppas equation in the following form:

log(M_t_/M_0_) = logk_m_ + nlogt (1)

In Equation (1), M_t_ is the amount of the drug released by the time t, M_0_ is the total amount of the drug entrapped in the carrier, k_m_ is the release rate constant calculated from the y-axis intercept, and n is the Korsmeyer–Peppas parameter calculated from the slope of the curve and is indicative of the mechanism of the release.

After 24 h of the release, a portion of the films was sampled out, dried and analyzed for surface morphology under a field-emission scanning electron microscope (FE-SEM). FE-SEM studies were carried out on an FEI Magellan 400 SEM operated at 30 kV voltage and 25 μA beam current. Sample preparation involved gluing the films onto clean aluminum stubs using carbon tape and subsequently sputter-coating them (Leica 600) with iridium up to a thickness of 3 nm to reduce surface-charging effects and the resulting artifacts. 

## 3. Results

The key hypothesis behind the concept tested in this study was that introducing redox-susceptible MnO_2_ nanoparticles to cast PCL films loaded with an insulin proxy would lead to structural deformations in the material under the reductive action exhibited by glucose, inducing drug release in the amount that is directly proportional to the concentration of glucose. To test this concept, the composite polymeric/inorganic films, i.e., MnO_2_@PCL, comprising MnO_2_ nanoparticles suspended in the PCL network loaded with methyl orange, were submerged into a release medium containing different concentrations of glucose. 

**Figure 2:**
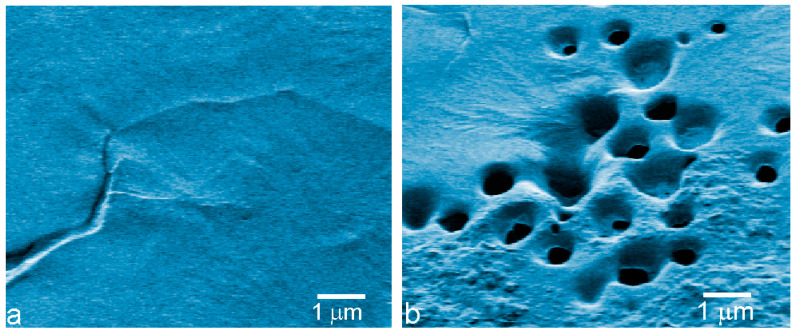
SEM images of the surfaces of PCL (**a**) and MnO_2_@PCL (**b**) thin films after exposure to the release medium supplemented with glucose for 24 h.

SEM images of the PCL and MnO_2_@PCL films after 24 h of release in a glucose-supplemented medium are shown in Figure 2a,b, respectively. Whereas the surface of the PCL films showed mild corrugations and crack formation, which opened up the structure and enhanced the drug release, the surface of the composite films showed characteristic pores formed in part by the sinking of the heavier MnO_2_ through the light polymeric film due to gravity and in part by the chemical changes caused by the reduction of MnO_2_ with glucose under alkaline conditions and the subsequent formation of metallic Mn in the system (Equation (2)). The transformation of MnO_2_ entailing the oxidation of glucose to gluconic (Equation (3)) and glucaric (Equation (4)) acids was likely to have been partial, given the moderate reductive activity of glucose, but still sufficient to cause pore formations causative of enhanced drug release. The role of hydrogen peroxide forming in the last step of the glucose-oxidation reaction (Equation (4)) is such that it may also contribute to the partial disintegration of the polymeric structure, facilitating pore-opening and the release of the drug. Given that the peroxide byproduct could also cause the acidification of the medium as per the H_2_O_2_ → 2H^+^ + O_2_ pathway, the alkalinity of the medium had to be relatively high. As two protons at most may form for each glucose molecule oxidized to glucaric acid, this would lead to a drop in the pH by a theoretical 1.5–2 units for the glucose concentration range utilized. Therefore, to ensure that the alkaline conditions facilitating the oxidation reaction were preserved throughout the reaction, the initial pH in the unbuffered system was set to 10. In fact, both of these byproducts of the oxidation of glucose, namely H_2_O_2_ production and acidification of the medium, have been used to create glucose-responsive drug delivery carriers [58].

Mn^4+^O^2−^_2_ → Mn^(0)^ + O_2_^(0)^ (2)

2H-(C=O)-(CHOH)_5_-H + O_2_ → 2H-(O=CH_2_)-(CHOH)_4_COOH (3)

2H-(O=CH_2_)-(CHOH)_4_COOH + 2O_2_ + 2H_2_O → 2HOOC-(CHOH)_4_COOH + 2H_2_O_2_ (4)

**Figure 3:**
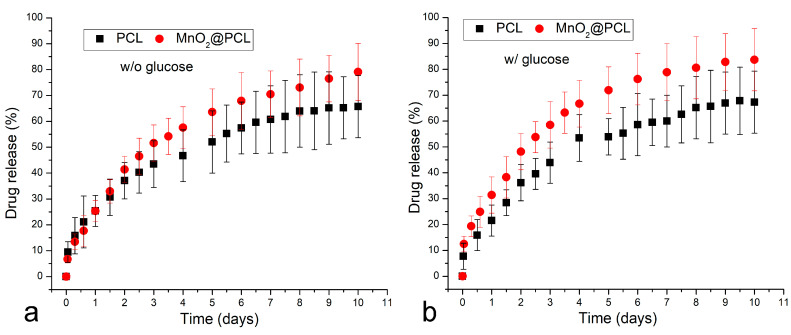
Comparative insulin proxy release profiles for PCL and MnO_2_@PCL thin films without (**a**) and with (**b**) 100 mg/mL glucose in the medium. Data points are shown as averages (n = 3), while error bars represent standard deviations.

**Figure 4:**
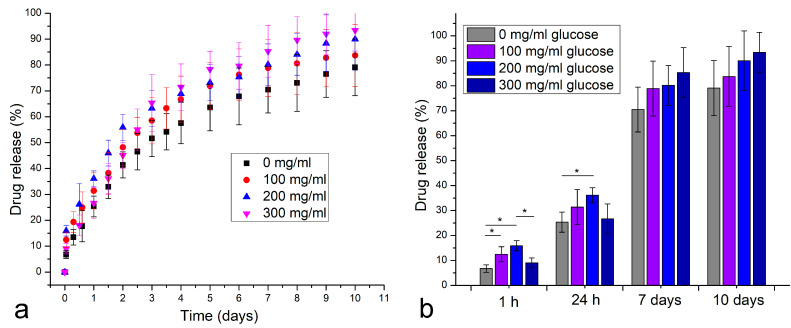
Comparative insulin proxy release profiles for MnO_2_@PCL films at different glucose concentrations in the medium (**a**) and the projected release after 1 h, 24 h, 7 days and 10 days at different glucose concentrations (**b**). Data points are shown as averages (n = 3), while error bars represent standard deviations. Statistically significantly (*p* < 0.05) different expressions between the sample groups are marked with asterisks.

As far as the release of the insulin proxy is concerned, it followed a characteristic profile resembling the first-order kinetics, where the release rate is directly dependent on the concentration of the drug inside the polymeric matrix. Because of the disruptions to this matrix caused by the presence of MnO_2_ nanoparticles, the release rate was higher when these nanoparticles were present in the matrix as compared with pure PCL films (Figure 3a), even when glucose was absent from the release medium. However, with the addition of glucose (Figure 3b), the rate of release of the insulin proxy visibly increased from the PCL films containing MnO_2_ nanoparticles, whereas the rate of the drug release from pure PCL films remained approximately the same as that when glucose was absent. Further, when the insulin proxy release profiles are compared for media containing incrementally higher concentrations of glucose (Figure 4a), it can be seen that the release rates steadily increase with the concentration of glucose in the medium. These trends confirm the ability of glucose to promote the release of the insulin proxy from the polymeric films enriched with redox-susceptible MnO_2_ nanoparticles. From Figure 4b, furthermore, it can be seen that the steadiest increase in the release rate with the concentration of glucose, applying across the entire 0–300 mg/mL range, occurred when the release process was performed in the order of days. In contrast, the most statistically significant increase, albeit within the 0–200 mg/mL glucose concentration range only, was detected when the release process was performed in the order of hours, which is the timescale of the greatest practical significance for application in the glucose-dependent delivery of insulin. 

**Figure 5:**
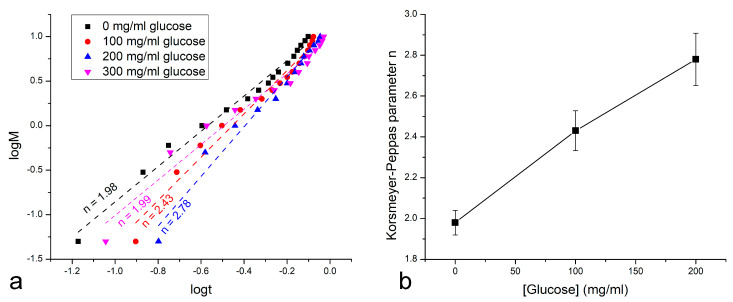
Korsmeyer–Peppas fits to the logarithmic functions of the degree of reaction completion, M, versus time for the release of the insulin proxy from MnO_2_@PCL films at different concentrations of glucose in the medium (**a**). Linear dependence of the release mechanism-indicative Korsmeyer–Peppas parameter n versus the concentration of glucose in the medium (**b**).

The insulin proxy release profiles were also analyzed for the mechanism of release based on the fits with the Korsmeyer–Peppas kinetic model. For the release from MnO_2_@PCL films, regardless of the concentration of glucose in the medium, the release mechanism, given the value of the Korsmeyer–Peppas exponent being higher than 1 (Figure 5a), was classified as non-Fickian super case II diffusion [59]. This type of diffusion defies Fick’s law owing to a nonlinear function of the mean squared displacement versus time and applies to a case where the diffusion is very fast compared with the polymer chain relaxation rate [60]. In the classical Fickian scenario and during case I transport, the polymer chains display relatively high mobility and the solvent penetrates them fairly easily, leading to subsequent drug diffusion. In contrast, the diffusion of the drug molecules during super case II transport precedes the restructuring of the relatively sluggish polymer upon swelling. It is very likely that this slow structural reorganization of the polymer due to interaction with the solvent enabled the reduction of the metal oxide phase to be the critical factor in causing this restructuring, along with the subsequently facilitated release of the drug. 

One effect favoring the observed enhancement of the insulin release rate in the presence of glucose may be tied to the semicrystalline nature of PCL. Namely, with the amorphous regions being more porous and containing an entropically greater number of degrees of freedom, they should represent the primary sites for the accommodation of the metal oxide precursors and the newly formed metallic nanoparticles. The typically enhanced degradation of these amorphous regions as compared with that of their crystalline counterparts [61] may contribute to the release of insulin and to the subsequent loosening and breaking of the more rigid crystalline chains.

Another interesting observation pertaining to the mechanism of the insulin proxy release is the monotonous increase in the Korsmeyer–Peppas parameter value with the increase in the glucose concentration in the 0–200 mg/mL range (Figure 5b). Notwithstanding that the release at the highest glucose concentration tested, namely 300 mg/mL, deviated from this trend, this still suggests an increasingly nonlinear and anomalous release accompanied by a higher diffusion rate at the expense of chain rigidity as the glucose concentration increases. This finding is well-supported by the trend in the absolute release profiles shown in Figure 4. Concordantly, all of the fits shown in Figure 5a were typified by r^2^ values higher than 0.965, although this value steadily decreased with the addition of glucose, from 0.986 for 0 mg/mL glucose to 0.976 for 100 mg/mL glucose to 0.975 for 200 mg/mL to 0.966 for 300 mg/mL, indicating a more disordered release as the conditions in the system became more reactive due to increasing amounts of reductive glucose in the system.

## 4. Discussion

These results combined largely support the concept of using redox-reactive metal oxide nanoparticles as drivers of structural changes in a polymer, allowing for the controllable release of an insulin proxy in direct proportion with the concentration of glucose in the medium. Regarding the mechanism of release, as per the central hypothesis of the study, glucose reduces Mn^4+^ ions contained within the MnO_2_ phase, forming partially zero-valent metallic Mn. With this phase conversion, the particles are likely to become smaller, not only because only one-third of the atomic constituents of MnO_2_ will end up in the solid Mn phase, but also because finer and more dissipative nanoparticles will become present in the system following the reduction process. In other words, glucose, in this case, can be thought of casually, not literally, as a “corrosive” that “eats away” the inorganic phase of the carrier, opening up the pores in the polymeric matrix and enabling a faster release of the drug, the rate of which increases in direct proportion to the glucose concentration. 

The concept, however, albeit preliminarily proven using a set of traditional characterization techniques, has a long way to go before becoming applicable in a real-life setting. A few inherent limitations of the model used here to set up and test this concept could be briefly mentioned as potential starting points for future refinements of the model and for bringing it closer to a medical application. 

One important limitation came in the form of the use of a small molecule dye, namely methyl orange, as a form of an insulin proxy, rather than insulin per se. To circumvent the unaffordable cost of pure insulin for the type of analyses reported here and also to satisfy the requirements of solubility inside the hydrophobic polymeric network, methyl orange was selected as an insulin substitute instead of a more hydrophilic proxy, such as, for example, rhodamine B [62]. The problem with this choice is that the fluorescent molecule of methyl orange does not only have an order of magnitude lower molecular weight than insulin, but is also considerably less hydrophilic than insulin. Namely, the partition coefficient of methyl orange (logP_oct/water_) reaches a maximal value of around 0.15 at the neutral pH and at more alkaline conditions, while it drops to near –1.0 at pH 2, when its sulfonate group is fully protonated [63]. Meanwhile, the logP_oct/water_ value of insulin can be estimated from the solubility values in water and n-octanol under the standard conditions to be around −2.4 [64]. The hydrophilicity of insulin, in fact, presents a major problem for its delivery across the intestinal epithelial barrier, for which reason it is often combined with various carriers that impart a degree of hydrophobicity to it [65], thus increasing its transportability via the oral delivery route. If insulin is incorporated into the PCL carrier in one such hydrophobic form, then the model implemented here would be a more veritable reflection of its release characteristics than it is for pure insulin. 

Next, two main properties of the model diverged from physiological conditions. First, as glucose oxidation requires an elevated pH, the pH of the medium in which the insulin proxy release took place was significantly higher than the physiological pH. The pH at which the structural changes in the drug delivery carrier were induced was, in fact, higher than that present in organs such as bile (pH 7.6–8.8) or pancreatic fluid (pH 8.8) [66], exceeding even that present during urinary tract infection (pH up to 9.1) [67], when urea in the urine undergoes urease-catalyzed hydrolysis to produce an abundance of ammonia. Second, the concentrations of glucose tested were two orders of magnitude higher than the physiologically relevant ones. Namely, blood sugar levels under 1 mg/mL under the fasting regimen or under 1.4 mg/mL under normal conditions are considered healthy; levels of 1–1.4 mg/mL under the fasting regimen and 1.4–2 mg/mL under normal conditions indicate a pre-diabetic state; levels higher than or equal to 1.4 mg/mL under the fasting regimen and higher than or equal to 2 mg/mL under normal conditions are indicative of diabetes. This concentration range spanning from 1 to over 2 mg/mL is 100 times lower than the 100–300 mg/mL range utilized in this study to overcome the weak propensity of glucose for oxidation in the absence of biological catalysts. The highest of the concentrations tested, i.e., 300 mg/mL, three times lower than the solubility of glucose in water under standard conditions [68], has been employed previously for a successful preparation of copper nanoparticles via the reduction of copper nitrate [69].

To end the discussion on a more positive note, a few aspects of the material reported here may be mentioned as starting points for imparting additional therapeutic modalities to it. For example, zero-valent Mn nanoparticles were used as peroxidase proxies for the colorimetric detection of hydrogen peroxide [70]. Considering that the gold standard principle behind the detection of glucose in virtually all commercial sensors is the conversion of H_2_O_2_ generated in the reaction between glucose and the enzyme glucose peroxidase to an electric current [71,72] via the standard Prussian Blue reaction at a transducer electrode [73], it is conceivable that the Mn component of the thin film synthesized and analyzed in this study could be used to add this sensory, diagnostic modality to its smart release properties. 

Next, it is worth recollecting that one of the key arguments in favor of smart, glucose-responsive release carriers of insulin lies in their ability to avoid the dangerous symptoms of hypoglycemia in patients with diabetes by ensuring that the concentration of insulin delivered is always adjusted to the glucose concentration in the tissue. Namely, because the physiological glucose concentrations in bodily fluids fluctuate hourly, any device releasing insulin at a constant rate, be it of the zeroth, first or any higher kinetic order, will run into a problem of inducing hypoglycemia whenever the released amount of insulin is excessive relative to the concentration of glucose in the tissue. Therefore, an ideal insulin delivery carrier must adjust its release kinetics to the glucose concentration in the tissue; in other words, it should be stimuli-responsive, that is, smart. Even then, however, conditions of hypoglycemia can naturally occur and it is tempting to consider that the concept tested here may not only be applicable in the mitigation of the symptoms of hyperglycemia, but that it may also be capable of alleviating the symptoms of naturally occurring hypoglycemia, another critical condition associated with diabetes. For this concept to work, the extra glucose from the medium could be captured by the material during its in vivo performance and then released when the glucose level in the blood drops below the 0.7 mg/mL threshold [74]. In analogy with another material that accomplished the effect of the controlled release of glucagon under the conditions of hyperinsulinemia [75], an aptamer or another ligand selectively binding insulin could be conjugated to glucagon and immobilized onto a polymer, so that, at a high insulin concentration in the medium, glucagon is released from the matrix through the competitive binding between free insulin and immobilized insulin, a concept similar in nature to the use of the transition between two manganese phases in this study to elicit the release of an insulin proxy. Other properties could also be appended to the material, considering that one major avenue for the future of medical devices lies in their hybrid, multifunctional capabilities for performance [76]. 

## 5. Conclusions and Future Perspectives

The susceptibility of manganese oxide nanoparticles to partial reduction with glucose was used to design a composite, polymeric/inorganic carrier for the smart delivery of an insulin proxy. The extent of the release of this small molecule compound was directly proportional to the concentration of glucose in the medium. The extent of this proportionality was dependent on the timescale of the process, in such a way that the steadiest increase in the release rate with the concentration of glucose was detected when the release process was performed in the order of days, whereas the most statistically significant increase was observed when the release process was performed in the order of hours. Future studies should focus on rendering the release process (i) feasible within the physiological pH range and (ii) sensitive to physiologically relevant glucose concentrations. Both of these effects are likely to be achievable with the use of molecular catalysts analogous to those operating under the physiological conditions in aerobic living organisms. These technical improvements of the fundamental new concept proposed and proven in this study may bring it closer to a real-life application for the mitigation of symptoms of hyperglycemia in patients with diabetes.

## Data Availability

Data will be available upon reasonable request.
